# Altered gut microbiota profile in patients with perimenopausal panic disorder

**DOI:** 10.3389/fpsyt.2023.1139992

**Published:** 2023-05-25

**Authors:** Shen Lin, Hongjin Wang, Jingjing Qiu, Minghong Li, Ebin Gao, Xiaofeng Wu, Yunxiang Xu, Guizhen Chen

**Affiliations:** ^1^Clinical Medical College of Acupuncture-Moxibustion and Rehabilitation, Guangzhou University of Chinese Medicine, Guangzhou, Guangdong, China; ^2^The Bao'an District TCM Hospital, The Affiliated Hospital of Guangzhou University of Chinese Medicine, Shenzhen, Guangdong, China; ^3^School of the Environment and Safety Engineering, Jiangsu University, Zhenjiang, Jiangsu, China

**Keywords:** perimenopause, panic disorder, gut microbiota, 16S rRNA, inflammation, tryptophan

## Abstract

**Introduction:**

Females in the perimenopausal period are susceptible to mood disorders. Perimenopausal panic disorder (PPD) is characterized by repeated and unpredictable panic attacks during perimenopause, and it impacts the patient's physical and mental health and social function. Pharmacotherapy is limited in the clinic, and its pathological mechanism is unclear. Recent studies have demonstrated that gut microbiota is strongly linked to emotion; however, the relation between PPD and microbiota is limitedly known.

**Methods:**

This study aimed to discover specific microbiota in PPD patients and the intrinsic connection between them. Gut microbiota was analyzed in PPD patients (*n* = 40) and healthy controls (*n* = 40) by 16S rRNA sequencing.

**Results:**

The results showed reduced α-diversity (richness) in the gut microbiota of PPD patients. β-diversity indicated that PPD and healthy controls had different intestinal microbiota compositions. At the genus level, 30 species of microbiota abundance had significantly different between the PPD and healthy controls. In addition, HAMA, PDSS, and PASS scales were collected in two groups. It was found that Bacteroides and Alistipes were positively correlated with PASS, PDSS, and HAMA.

**Discussion:**

Bacteroides and Alistipes dysbiosis dominate imbalanced microbiota in PPD patients. This microbial alteration may be a potential pathogenesis and physio-pathological feature of PPD. The distinct gut microbiota can be a potential diagnostic marker and a new therapeutic target for PPD.

## Introduction

Perimenopausal panic disorder (PPD) is characterized by unexpected and repeated panic attacks, severe sensations of impending death, and autonomic nervous system dysfunction, including palpitations, tremors, and sweating (1, 2). A recent cross-national epidemiology study indicates that the panic attack incidence and lifetime prevalence rate of panic disorder are 13.2 and 1.7%, respectively (3). Female reproductive cycles often influence the course of panic disorder (PD) that worsens with menopause (4). Individuals aged between 40 and 49 years have a 3.3% prevalence rate of PD, of which 2.4% are in women (5). As life expectancy rises, the incidence of PPD elevates, causing more medical burdens and lower quality of life. Cognitive behavioral therapy (CBT) combined with first-line drugs has significant clinical short-term effects but limited long-term effects. In addition, several adverse effects and high recurrence rates still exist (6, 7). The pathogenesis of PPD includes brain network function dysfunction, neurotransmitter disturbance, microbiota disorders, and reproductive endocrine dysregulation (8). However, the detailed mechanism remains unclear. Therefore, it is important to explore the pathogenesis of PPD for prevention and treatment strategies.

Recent research implicates the gut microbiota in mental health and mood disorders, including anxiety, depression, bipolar, and stress disorders (9). A disturbed balance of intestinal microbes can cause the loss of several host functions, including neurotransmitter dysbiosis, immunological dysfunction, and increased blood–brain barrier permeability (10). Several studies show that the intestinal microbiota has modulated the central nervous system (CNS), such as the amygdala, hippocampus, and prefrontal cortex (11, 12). These brain areas are the neuroanatomical basis of panic. Additionally, the deficient estrogen alters intestinal microbiota balance during perimenopause (13) and induces anxiety-like behavior (14). Therefore, we believe the intestine microbial imbalances of perimenopause might cause panic attacks.

To the best of the authors' knowledge, there is no study on the gut microbiota of perimenopausal panic disorder. Therefore, this study examined 16S rRNA sequencing results to understand the gut microbiota composition in PPD patients. It was hypothesized that PPD patients had different intestinal microbiota, including microbial diversity, relative abundance, and taxonomic composition, compared to matched healthy controls. Furthermore, the gut microbe and panic symptoms scale were investigated for associations. These results provide evidence for the pathological mechanism, diagnosis, and treatment of PPD.

## 2. Methods

### 2.1. Patient recruitment

This study recruited patients with PPD from the Menopausal Syndrome Specialist Clinic in the Bao'an District TCM Hospital (Shenzhen, China). All PD patients aged between 41 and 60 years were diagnosed according to the DSM-5 and their HAMA score was ≥ 14 (2, 15). The exclusion criteria were somatoform disorders or physical diseases causing PD, malignant tumors, or antipsychotic drug use. People who had used probiotics, antibiotics, or other drugs affecting the intestinal microbiota within the last 3 months before sampling were excluded from the study (16, 17). Healthy women matched for age were also engaged in the control group. All patients provided basic demographic information. The Hamilton Anxiety Scale (HAMA), Panic-associated Symptoms Scale (PASS), and Panic Disorder Severity Scale (PDSS) were used to score the test for anxiety and panic symptoms. In general, ~4–6 g fecal samples from PPD patients or healthy controls were collected once at the start of the experiment. Next, the samples were placed in a sterile tube and frozen at −80°C until they were sequenced. Each participant signed an informed consent form, stating that they understood the risks and benefits of having their feces used for scientific research.

### 2.2. 16S high-throughput sequencing

The tiny subunit of bacterial ribosomes contains 16S rRNA, a nucleic acid sequence that indicates biological species and is the primary indicator of bacterial phylogeny and taxonomic identification. The 16S rRNA Amplicon Sequencing involves one or more variant regions and utilizes the conserved regions for polymerase chain reaction (PCR) amplification. The sequencing examination and identification of the high variant regions of microorganisms' strains have become an important approach for analyzing their composition and structure. Small fragment libraries are constructed and sequenced using paired-end sequencing on the Illumina MiSeq sequencing platform. The species annotation and relative abundance are determined by splicing and filtering reads and clustering operational taxonomic units (OTU). Analyses of the alpha diversity (α-diversity) and the beta diversity (β-diversity) can investigate the distinctions between samples.

### 2.3. Experimental procedure

This study extracted the microbial genomic DNA from feces using QIAamp^®^ DNA Stool Mini Kit (Qiagen, German). The OD-1000 spectrophotometer (OneDrop, Nanjing, China) and 1% agarose gel electrophoresis measured DNA concentration and purity. Diluted genomic DNA was used as a template. The gene was amplified efficiently and accurately by PCR with GoTaq^®^ Hot Start Colorless Master Mix (Promega, USA) using the V4 region of 16S rRNA primers with Barcode (515F-806R). The concentrations of PCR products were measured with Pico Green assay and then mixed at the same concentration. PCR Purification Kit (Qiagen) was applied to purify and recover PCR-amplified products. In this study, the purified and recovered products were mixed using Illumina-compatible primers in a second round of PCR amplification. Pico Green fluorescently quantified the second-round products, Agilent 2200 TapeStation, and Illumina MiSeq platform (Illumina, USA), analyzed and sequenced them, respectively.

### 2.4. Preprocessing of sequencing data

Raw data from the Illumina MiSeq platform provided low-quality information that could impede processing. Consequently, it was preprocessed before the analysis, with the following steps. (i) The raw data from high-throughput Sequencing was isolated from the individual sample data. Then, a quality check was performed following the barcode information and primer sequences. (ii) Each 16S rRNA gene sequence was aligned using the Ribosomal Database Project (RDP) Classifier software (version 2.3) and the Silva database. Next, sequence categorization levels (phylum, class, order, family, and genus) were determined. (iii) Sequences were classified into OTUs based on sequence similarity of 97% using the Mothur software. Then, sequence numbers were used to determine OTU abundance.

### 2.5. Statistical analysis

This study used Illumina MiSeq to calculate the diversity levels within and between communities (alpha and beta diversities, respectively). MetaboAnalyst software was used to depict and compare the differences in a microbial population with principal component analysis (PCA) and orthogonal partial least squares-discriminant analysis (OPLS-DA). Statistical analysis was performed using SPSS software (version 23.0). The Shapiro–Wilk test assessed the normality of the data. An unpaired *t*-test compared between-group differences if the data were a normal distribution. The non-normal data were analyzed by the non-parametric Mann–Whitney U-test. According to the normality of the data, the Pearson or Spearman correlations were conducted to assess the PASS, PDSS, and HAMA Scales with gut microbiota, and *p* < 0.05 was considered statistically significant. Differentially abundant taxa were analyzed using the LEfSe (α = 0.05, effect size threshold of 2). The non-parametric factorial Kruskal–Wallis sum-rank test determined and identified the taxa whose abundance characteristics differed significantly, and LDA analysis evaluated species abundance on the differential effect.

## 3. Results

### 3.1. Participants' characteristics

This study engaged 80 women (40 in each PPD and control group). There were no variations in age (PPD = 49.85 ± 4.12 years vs. Con = 51.65 ± 4.83 years, *p* = 0.077) and height (PPD = 158.9 ± 3.54 cm vs. Con = 159.4 ± 4.67 cm, *p* = 0.610) between PPD and healthy control group. However, PPD patients were higher weight (PPD = 56.30 ± 4.45 kg vs. Con = 54.15 ± 3.67 kg, *p* = 0.021) and body mass index (BMI) (PPD = 22.29 ± 1.48 kg/m^2^ vs. Con = 21.34 ± 1.50 kg/m^2^, *p* = 0.006) than healthy control group. PPD patients had a higher risk of gaining weight, consistent with a previous study on PD (18). The PDSS and PASS scores were 9.20 ± 3.24 and 10.63 ± 3.72 in PPD patients, respectively, and the HAMA scale was different between the two groups (PPD = 28.48 ± 7.15 vs. Con = 5.83 ± 2.34, *p* < 0.05), indicating that the PPD patients suffered from panic or anxiety (Table 1; Supplementary Figure 1).

**Table 1:** Demographic characteristics of the participants.

	**Controls (*n* = 40)**	**PPD (*n* = 40)**	**P value**
Age (years)	51.65 ± 4.83	49.85 ± 4.12	0.077^a^
Height (cm)	159.4 ± 4.67	158.9 ± 3.54	0.610^a^
Weight (kg)	54.15 ± 3.67	56.30 ± 4.45	0.021^a^
BMI (kg/m^2^)	21.34 ± 1.50	22.29 ± 1.48	0.006^a^
**BMI distribution [*****n*** **(%)]**			
Underweight	1 (2.5%)	0 (0%)	
Normal	48 (95%)	49 (97.5%)	
18.5–21.7	23 (57.5%)	15 (37.5%)	
21.7–24.9	15 (37.5%)	24 (60%)	
Overweight	1 (2.5%)	1 (2.5%)	
Education (%, *n*)			0.811^c^
Journal high school and below	2 (5%)	3 (7.5%)	
Technical and senior high school	14 (35%)	15 (37.5%)	
Journal college school and above	24 (60%)	22 (55%)	
Job (%, *n*)			0.777^c^
Farmer	1 (2.5%)	2 (5%)	
Medical	2 (5%)	0 (0%)	
Housewife	8 (20%)	10 (25%)	
Worker	5 (12.5%)	8 (20%)	
Administrative staff	4 (10%)	3 (7.5%)	
Teacher	5 (12.5%)	3 (7.5%)	
Other	15 (37.5%)	14 (35%)	
Smoking (%, *n*)			0.479^b^
Yes	3 (7.5%)	5 (12.5%)	
No	37 (92.5%)	35 (87.5%)	
Drinking (%, *n*)			0.608^b^
Yes	1 (2.5%)	3 (7.5%)	
No	39 (97.5%)	37 (92.5%)	
**Scale score**			
PDSS	–	9.20 ± 3.24	–
PASS	–	10.63 ± 3.72	–
HAMA	5.83 ± 2.34	28.48 ± 7.15	< 0.05^d^

Data are presented as Mean ± SD (standard deviation); ^a^Independent-samples T test; ^b^Yates's correction for continuity; ^c^Fisher's exact test; ^d^Mann–Whitney U;

PPD, Perimenopausal panic disorder; BMI, Body mass index; Underweight, BMI < 18.5; Normal weight, BMI 18.5–24.9; Overweight, BMI 25-29.9; PDSS, Panic Disorder Severity Score; HAMA, Hamilton Anxiety Scale Score; PASS, Panic Associated Symptoms Score.

### 3.2. Microbiota diversity in PPD patients

**Figure 1:**
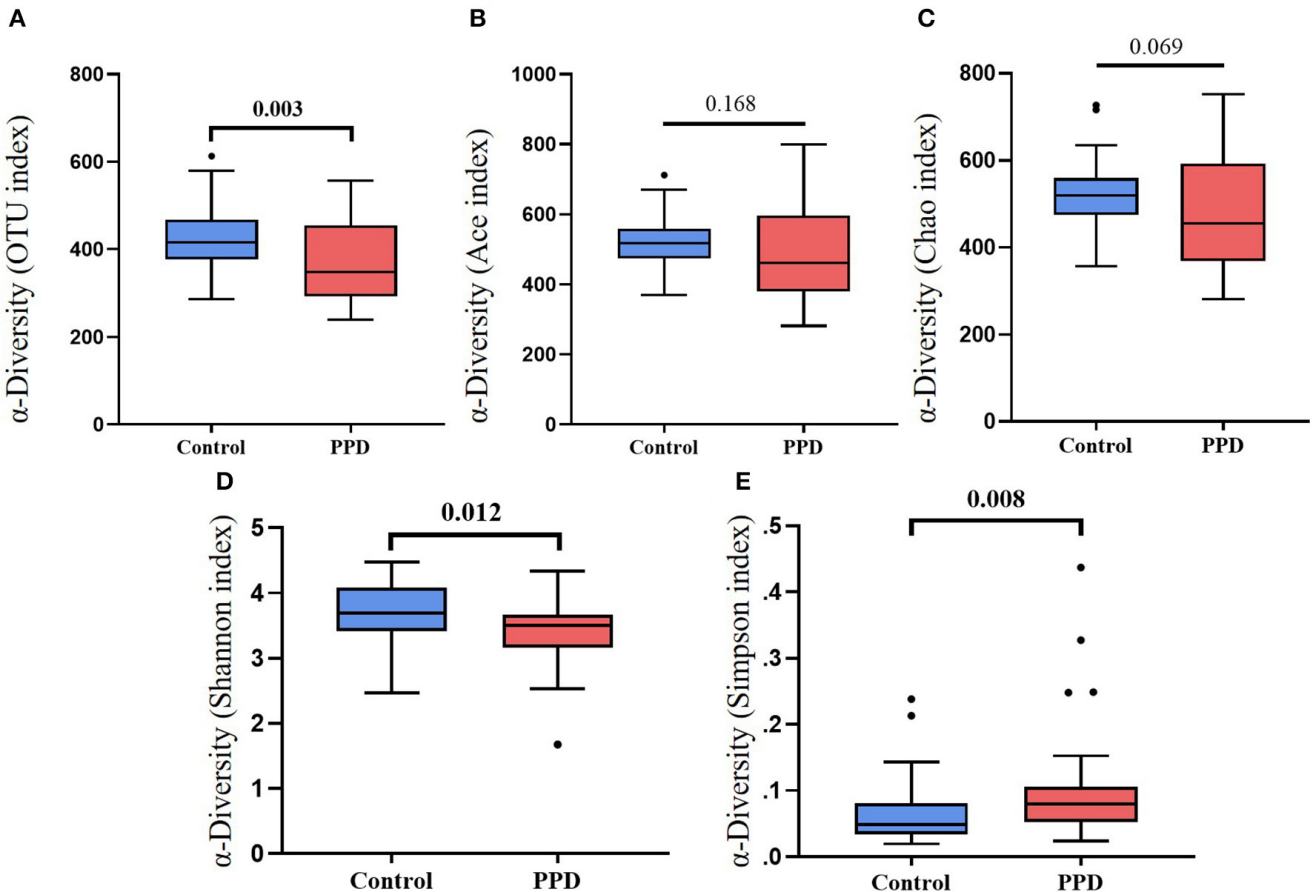
Box plots of α-diversity between PPD patients and healthy controls. **(A)** OTU index, *p* = 0.003. **(B)** ACE index, *p* = 0.168. **(C)** Chao index, *p* = 0.069. **(D)** Shannon index, *p* = 0.012. **(E)** Simpson index, *p* = 0.008.

**Figure 2:**
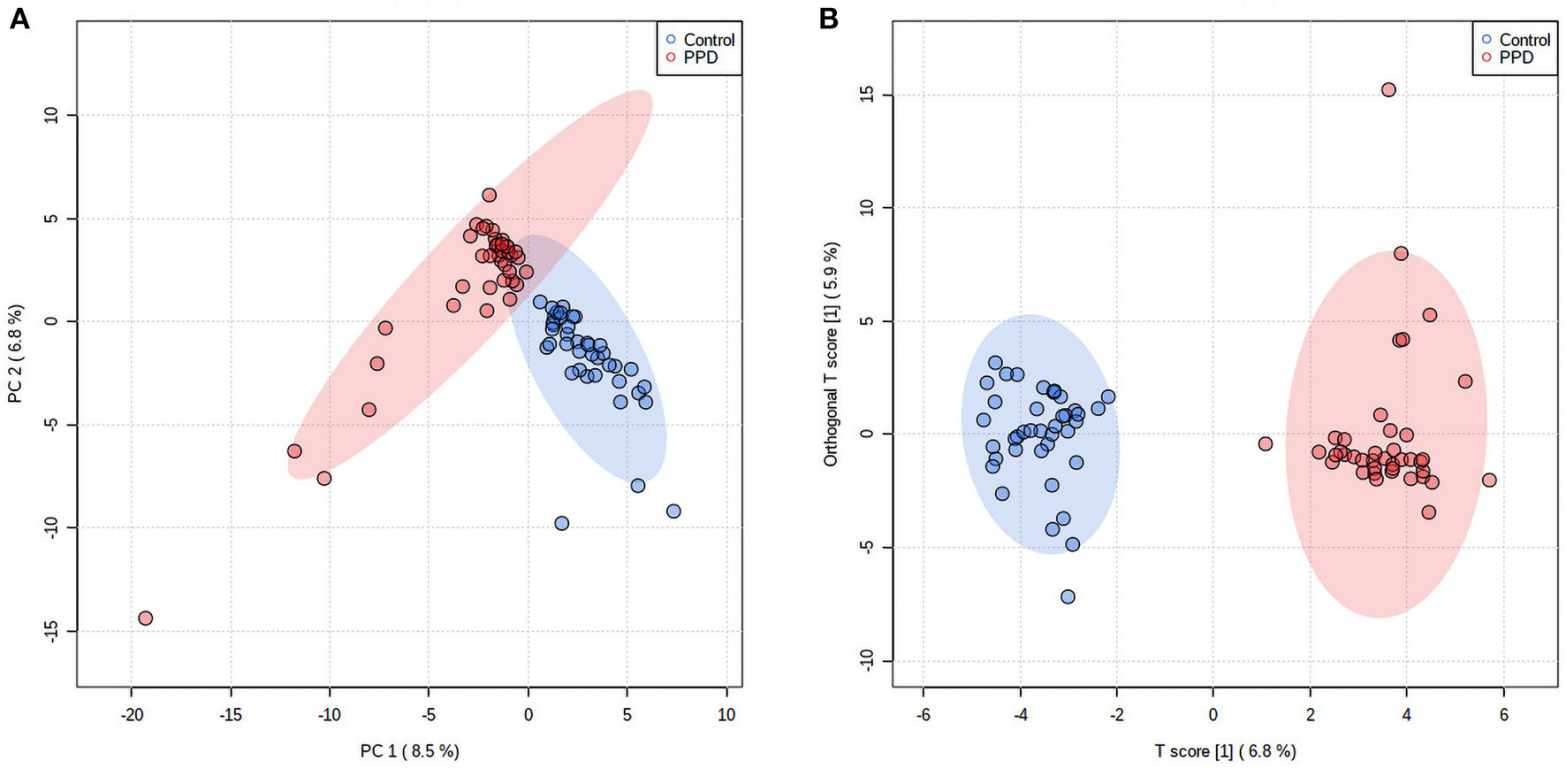
Difference in gut microbiota composition in PPD patients and healthy controls. Each point represents the microbiota of each subject, and each group of subjects was labeled with a different color. The ellipses display 95% confidence regions. **(A)** PCA, PC1 = 8.5%, PC2 = 6.8%. **(B)** OPLS-DA, T score = 6.8%, Orthogonal T-score = 5.9%.

Alpha diversity, including the OTU index, ACE, Chao index, Shannon, and Simpson suggested the abundance and diversity of gut microbiota. In this study, alpha diversity was richer and more diverse in gut bacteria species in healthy controls than in PPD patients (Figure 1). PCA and OPLS-DA measured β-diversity (between-habitat diversity) (Figure 2), and the abundance and dispersion of gut microbiota were compared by matrix data. PCA is an unsupervised method that performs ANOVA on several samples and dimensionality reduction in multidimensional data. PCA discovered the directions that best explained the variance in a dataset and represented sample differences on the two-dimensional coordinate map. The principal component 1 (PC1) and principal component 2 (PC2) were 8.5% and 6.8%, respectively (Figure 2A). The OPLS-DA is a supervised modeling method that distinguished variations in a dataset relevant to predicting group labels by dimension reduction. The horizontal axis and the abscissa direction represented the score value of the main component and the difference between groups, respectively. The vertical axis and the ordinate represented the orthogonal component value and the difference within the group, respectively (Figure 2B). A different group of gut microbiota was split into groups and clustered accordingly.

### 3.3. Difference in gut microbiota between PPD patients and healthy controls

**Figure 3:**
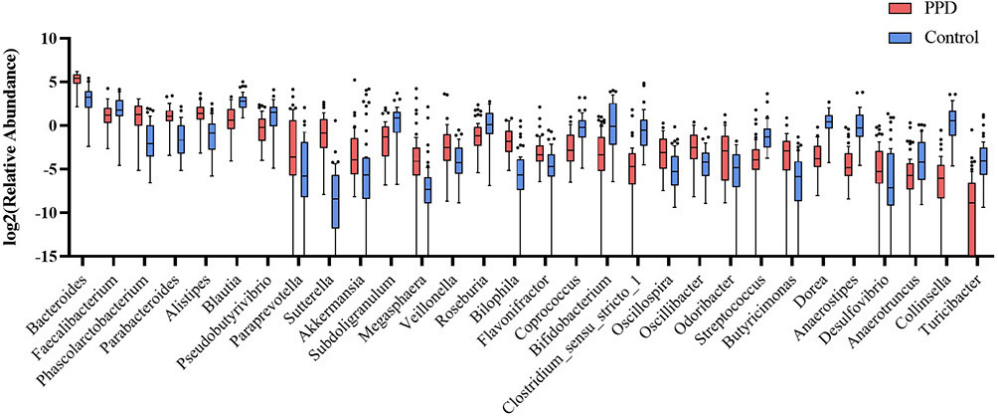
Comparison of the mean abundance of the top 30 significant dominant and specific bacterial genera in the PPD patients and healthy controls. Red, healthy controls. Blue, PPD patients.

The study classified the fecal samples into 1580 OTUs after analysis. A percent stacked column chart was used to visualize gut microbiota abundance. The results showed that the phylum Bacteroidetes and Verrucomicrobia were more abundant, and the Firmicutes and Actinobacteria were scarcer in PPD patients than in healthy controls (Supplementary Figure 2; Supplementary Table 1). The top 60 genera accounted for over 99% of the microbiota among all samples (Supplementary Figure 3; Supplementary Table 2). The study used cluster analysis and heatmaps to determine the relative abundance of genera between samples (Supplementary Figure 4). PPD patients had a microbiota abundance that contained significantly greater amounts of Bacteroides, Phascolarctobacterium, Parabacteroides, Alistipes, Paraprevotella, Sutterella, Akkermansia, Megasphaera, Veillonella, Bilophila, Flavonifractor, Oscillospira, Oscillibacter, Odoribacter, Butyricimonas, and Desulfovibrio than the healthy controls. In contrast, the healthy controls had significantly higher contents of Faecalibacterium, Blautia, Pseudobutyrivibrio, Subdoligranulum, Roseburia, Coprococcus, Bifidobacterium, Clostridium_sensu_stricto_1, Streptococcus, Dorea, Anaerostipes, Anaerotruncus, Collinsella, and Turicibacter than the PPD patients (Figure 3).

**Figure 4:**
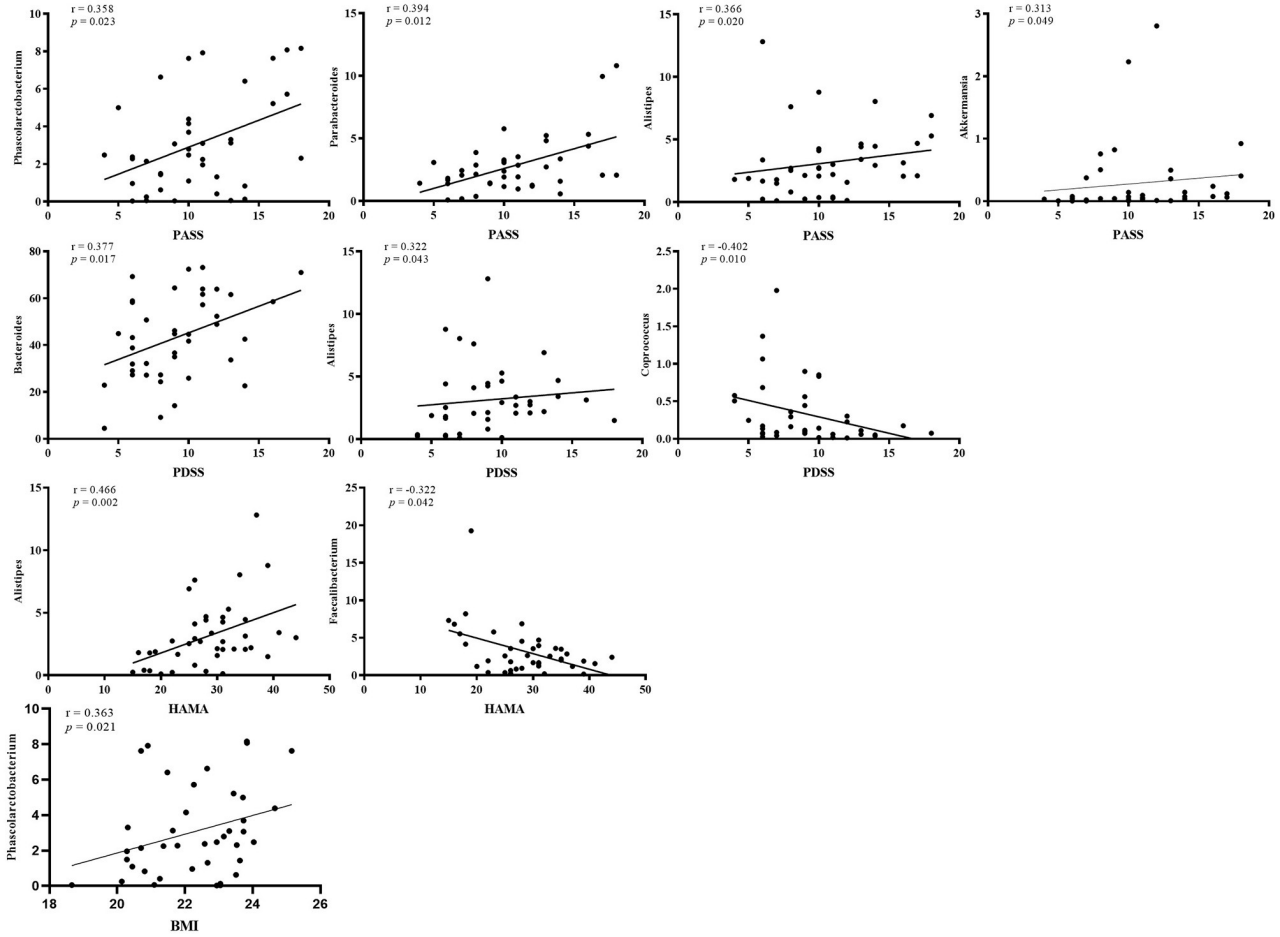
Correlation analysis between microbiota at the genus level and PASS, PDSS, HAMA scores, and BMI in PPD. PASS, panic-associated symptoms scale; PDSS, panic disorder severity scale; HAMA, Hamilton anxiety scale; BMI, body mass index.

**Figure 5:**
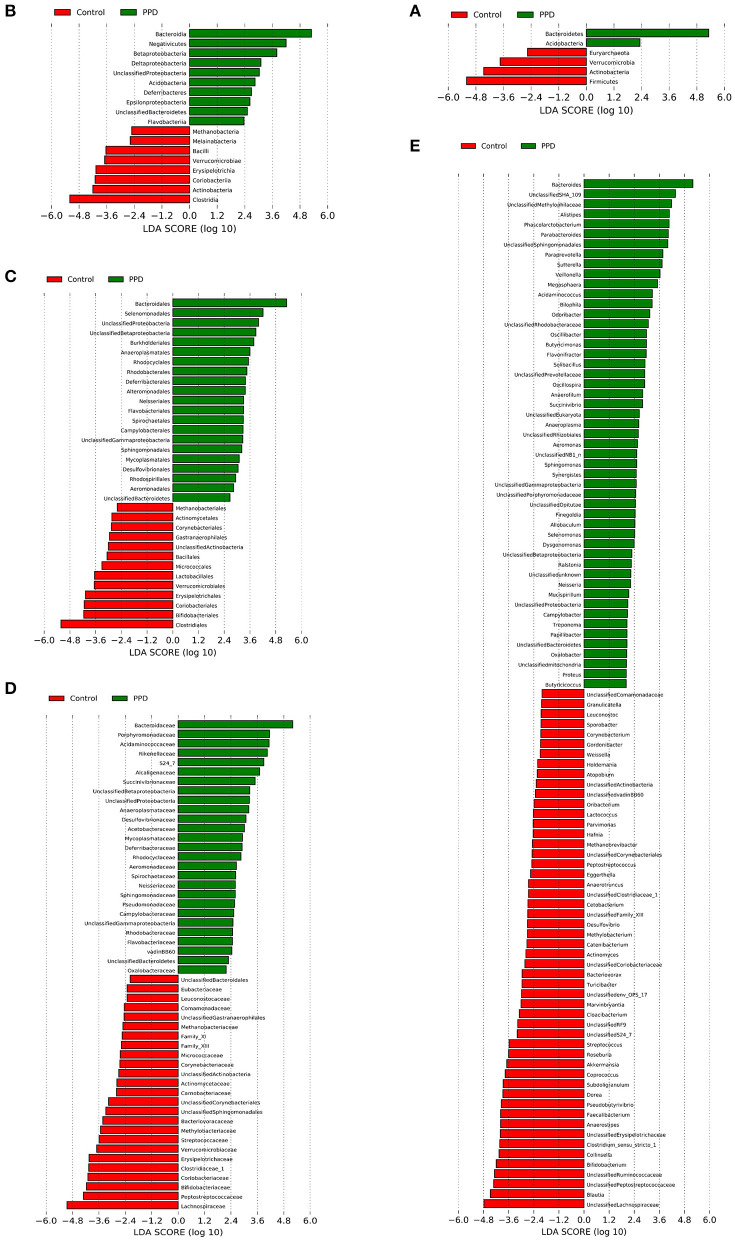
LEfSe analysis revealed diverse intestinal flora taxa and species between PPD patients and healthy controls (LDA score > 2, *p* < 0.05). **(A–E)** Classification levels of phylum, class, order, family, and genus. The graph was generated using the LEfSe program. Red, healthy control. Green, PPD patients.

A Spearman correlation analysis showed that the PASS was positively correlated with Phascolarctobacterium (*r* = 0.358, *p* = 0.023), Parabacteroides (*r* = 0.394, *p* = 0.012), Alistipes (*r* = 0.366, *p* = 0.02), and Akkermansia (*r* = 0.313, *p* = 0.049) at the genus level. The Bacteroides (*r* = 0.377, *p* = 0.017), Alistipes (*r* = 0.322, *p* = 0.043), and Coprococcus (*r* = -0.402, *p* = 0.01) were related with PDSS. The HAMA scales were positively correlated with Alistipes (*r* = 0.466, *p* = 0.002) and negatively correlated with Faecalibacterium (*r* = −0.322, *p* = 0.042). The Phascolarctobacterium (*r* = 0.363, *p* = 0.021) was positively correlated with BMI in PPD (Figure 4; Supplementary Table 3). The LEfSe approach identified major gut microbiota species from phylum to genus between healthy control and PPD patients, according to the LDA scores (*p* < 0.05, LDA score > 2) (Figure 5).

## 4. Discussion

This research is the first to identify significant distinctions in gut microbiome between perimenopausal panic disorder (PPD) patients and healthy women (healthy controls).

Gut microbiota has been reported extensively in mood disorders. However, research on it related to PPD is limited. Recent research has confirmed that gut microbial imbalance is one of the etiologies in psychiatric diseases (19, 20). Studies on animals have found that microbiota alteration can influence anxiety-like and despair behavior in stress-induced mice (21, 22). PD patients have a higher abundance of Bacteroidetes in oral microbiota than healthy controls (23). This study found that PPD patients and healthy controls had distinct significant differences in gut microbiota. Community richness (ACE Index and Chao Index) had no difference between PPD and healthy women, while evenness (Simpson Index) was higher in PPD. The β-diversity showed different microbiota communities in PPD patients compared to healthy control, and the microbial composition of PPD had changed, as reported in prior literature (24, 25). In addition, PPD patients had higher Bacteroidetes and the ratio of Bacteroidetes/Firmicutes was increased. The same alteration in gut microbiota was also found in generalized anxiety disorder (GAD) patients (25). These results suggest that the gut microbial balance of PPD patients was disorder. Dysbiosis of gut microbiota can induce anxiety or fear in the central nervous system (CNS) through aberrant immunological responses (26, 27). A study has shown that pro-inflammatory cytokines are also high levels in panic disorder (28). Estrogen inhibits inflammatory mediator production in the brain (29). Estrogen deficiency in perimenopause increases the pro-inflammatory cytokines that affect neurological systems (30, 31). The reduced pro-inflammatory cytokines by probiotic therapy can alleviate stress and anxiety (32–34). Therefore, the perimenopausal panic states might be induced by microbiota imbalance, causing an inflammatory immune response.

16S rRNA is important for assessing the physio pathology characteristics of PPD patients' gut microbiota. Although an interaction between microbiota and anxiety is recognized, there is less evidence of the specific microbiota of panic and their interactions. Prevotella and Veillonella are higher in panic oral microbiota and might influence CNS by increased inflammation (23). In the present research, PPD patients had more abundances of Bacteroides and Alistipes in the intestinal tract than healthy controls, and the two bacteria were positively correlated with panic and anxiety symptoms. In the literature, Bacteroides and Alistipes are positively correlated with anxiety in GAD patients (24, 25). In addition, Alistipes can influence the tryptophan metabolism and cause serotonergic system imbalance (35, 36). This imbalance can modify neurological impulses related to anxiety (37). Low tryptophan availability can inhibit the generation and release of serotonin in the brain, causing panic or anxiety (38).

Excessive Bacteroides and Alitipes can cause inflammation (35, 39). The pro-inflammatory cytokine can activate indolamine 2,3-dioxygenase (IDO), transforming tryptophan into kynurenine (40). Kynurenine can cross the blood–brain barrier to the brain easily and produces catabolite, Quinolinic acid, and Kynurenic acid (41). The Quinolinic acid has excitability and neurotoxicity (42) and induces an anxiogenic effect (43, 44), while the Kynurenic acid has neuroprotective effects (45). Moreover, IDO enzymes are more susceptible to immune activation in females than males and increase anxiogenic tryptophan catabolite levels (46). Variations of tryptophan pathway metabolism cause Bacteroides and Alistipes to influence PPD (47–49). Therefore, the Bacteroides and Alistipes can be biomarkers in PPD and may influence panic symptoms by regulating the immune–tryptophan–kynurenine pathway.

Furthermore, the gut microbiota can influence small molecule metabolites. Indoles and short-chain fatty acids (SCFAs) are important immune response modifiers and can exert anti-inflammatory effects (50). The aryl hydrocarbon receptor can be activated by indole metabolites, which can induce regulatory T-cell differentiation and release anti-inflammatory cytokines (51). In addition, SCFAs are endogenous and decrease IL-1β, IL-6, and TNF-α levels during host inflammatory response (52). SCFAs mediate systemic inflammation and central neuroimmune function. SCFAs have neuroactive properties and may promote serotonin biosynthesis in the brain, alleviate panic disorder (53, 54). Butyrate, as an anti-inflammatory SCFAs, can inhibit inflammatory responses (55) and indirectly mediate brain functions by stimulating the vagus nerve and regulating immune responses (56). The current study demonstrated that the Faecalibacterium, Coprococcus, and Roseburia are higher in healthy controls than in PPD patients. These three colonies of microbiota can produce butyrate (57, 58). Faecalibacterium as a novel probiotic therapy plays a role in low-grade inflammation (59). In addition, PPD patients have higher Phascolarctobacterium and Parabacteroides, which can metabolize amino acids and proteins, produce toxic substances, such as ammonia, putrescine, and phenol (60). Similar outcomes have been observed in major depression disorder (61). Although there is a significant difference in BMI values between the groups, the majority of both PPD patients and healthy controls fall within the normal range of BMI (< 24.9) in our study. Hence, we propose that the increased abundance of Phascolarctobacterium in PPD patients may be a potential factor contributing to their higher BMI. Additionally, increasing Roseburia and decreasing Parabacteroides are correlated with healthier eating behavior (62), which supports demographic results that PPD patients have higher BMI and weight. Therefore, the small molecule metabolites modulated by microbiota may further influence anxiety and panic symptoms. The gut microbiota may impact perimenopausal panic symptoms by regulating host immunity, metabolism, and inflammation.

Women are twice likely to develop affective disorders than males. Anxiety symptoms are observed more frequently in the menopause transition (63). A systematic review indicated that the estradiol variation could cause different gut microbiota between premenopausal and postmenopausal states (64). Female hormones play an important role in gut microbiota composition (13). Postmenopausal women treated with estrogen-like compounds isoflavones can suppress Clostridiaceae which is related to inflammation (65, 66). The gut microbiota impacts estrogen levels by secretion of β-glucuronidase deconjugating estrogen (67). Reduced gut microbiota diversity can decrease estrogen metabolism (68), decrease the glucocorticoid neutralization effect, and make women more vulnerable to stress (69). A study on animals documented that female mice with gut microbiota variations were more susceptible to stress (70). It is because menopause women are more susceptible to exogenous stress, which can activate the HPA axis to increase cortisol and change in gut microbiota (71, 72). Therefore, further study should be conducted on the mechanism of intricate intestinal microbial interactions with perimenopausal panic disorder.

This study focused on the difference in the gut microbiota between PPD patients and healthy controls. The Bacteroides and Alistipes abundances are highly correlated with perimenopausal panic symptoms and can represent diagnostic biomarkers for PPD patients. A microbiota imbalance can induce neuroinflammation, neurometabolic dysregulation, and neuroimmune dysfunction, leading to a panic attack.

The current study has some limitations. This study recruited PPD patients from only one city, Shenzhen (China). The result might vary if respondents with different dietary, climates, and geography are studied. Geographical factors more significantly impact microbial variance than dietary and urbanization factors (73). Second, this study's sample is relatively small and, thus, does not strongly represent diversity well. Future studies need to consider larger datasets with greater geographical coverage. Lastly, this study lacks the results of gut microbiota in female reproductive cycles except for the perimenopausal period. The effect of estrogen on intestinal microbiota is unclear and can be improved in subsequent studies. This study indicated that gut microbiota in PPD is important to immune response. A follow-up study will be conducted to investigate the effects further.

## 5. Conclusion

We discovered that intestinal flora is different between PPD and healthy women. Bacteroides and Alistipes dysbiosis dominate imbalanced microbe in PPD. The gut microbiota can influence PPD through immune inflammation and tryptophan–kynurenine pathway metabolism. This microbial alteration may be a potential pathogenesis and physio-pathological feature of PPD. The findings of this research will serve as a supporting evidence for the clinical diagnosis and therapy of PPD. Future study should investigate the in-depth interaction between PPD and gut microbiota and optimize the treatment ideas.

## Data availability statement

The original contributions presented in the study are included in the Supplementary material, further inquiries can be directed to the corresponding authors.

## Ethics statement

The studies involving human participants were reviewed and approved by Ethics Committee of Affiliated Bao'an TCM Hospital of Guangzhou University of Chinese Medicine (No. 20140227-2) and the Chinese Clinical Trial Registry, ChiCTR-INR-16009724. The patients/participants provided their written informed consent to participate in this study.

## Author contributions

YX and GC obtained funding, conceptualized the study, and designed the protocol. SL supervised recruitment, analyzed data, and drafted the manuscript. HW, JQ, and ML recruited and coordinated the trial and manuscript revision. EG completed sample sequencing and reviewed the manuscript. XW supported recruitment and sample collection. All authors provided a revision of the manuscript and approved the final manuscript.

## Supplementary material

Supplementary Figure 1 Subject inclusion flow chart.

Supplementary Figure 2 Stacked bar plots showing the distribution of taxa in PPD patients and healthy controls. Relative abundance at the phylum level.

Supplementary Figure 3 Stacked bar plots showing the distribution of taxa in PPD patients and healthy controls. Relative abundance at the genus level.

Supplementary Figure 4 Cluster analysis and heatmap of top 50 species abundance at the genus level in PPD patients and healthy controls. The clustering result is shown in the form of a dendrogram and heatmap. Metaboanalyst (version 5.0) was used for clustering. Each column represents the analyzed samples, and each row represents one of the top 50 microbiota genera. On top of the heatmap: red squares, healthy controls; green squares, PPD patients. The dendrogram for samples is shown on top of the heatmap, and the microbiome dendrogram is on the left side. Dark blue to dark red color gradient denotes lower to higher expression.

Supplementary Table 1 Top five phyla microbiota between PPD patients and healthy controls. Median (IQR: 25, IQR: 75).

Supplementary Table 2 Top 60 genera microbiota between PPD patients and healthy controls.

Supplementary Table 3 Correlation analysis between BMI, PASS, PDSS, HAMA scales, and fecal microbiota.
