# Supplementary figures and images for: Altered gut microbiota profile in patients with perimenopausal panic disorder
Source: Front Psychiatry. 2023 May 25;14:1139992. doi: 10.3389/fpsyt.2023.1139992 (PMC10249373; doi:10.3389/fpsyt.2023.1139992)

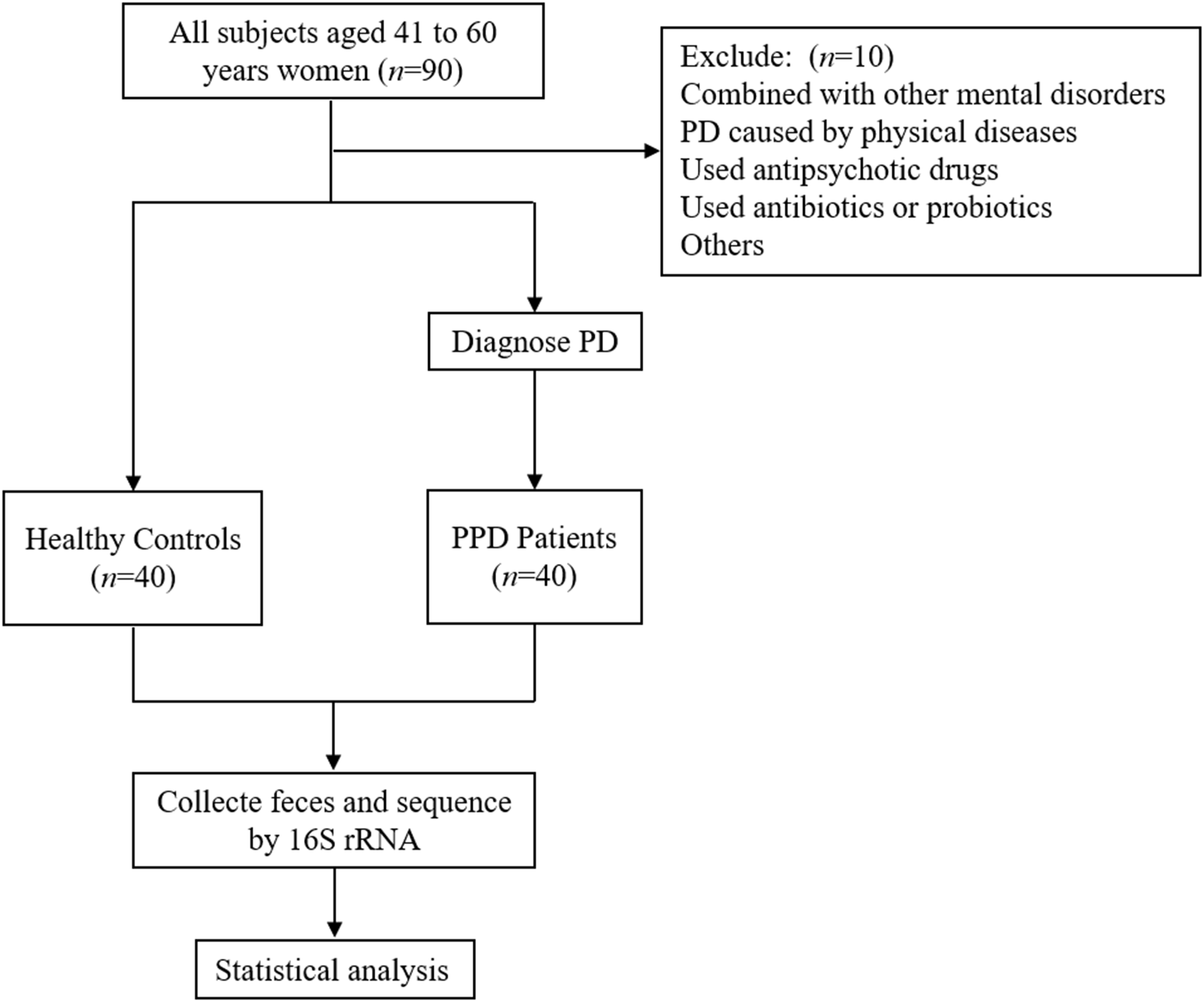

Supplement: Supplementary Figure 1 — Subject inclusion flow chart. [file Image_1.PNG]

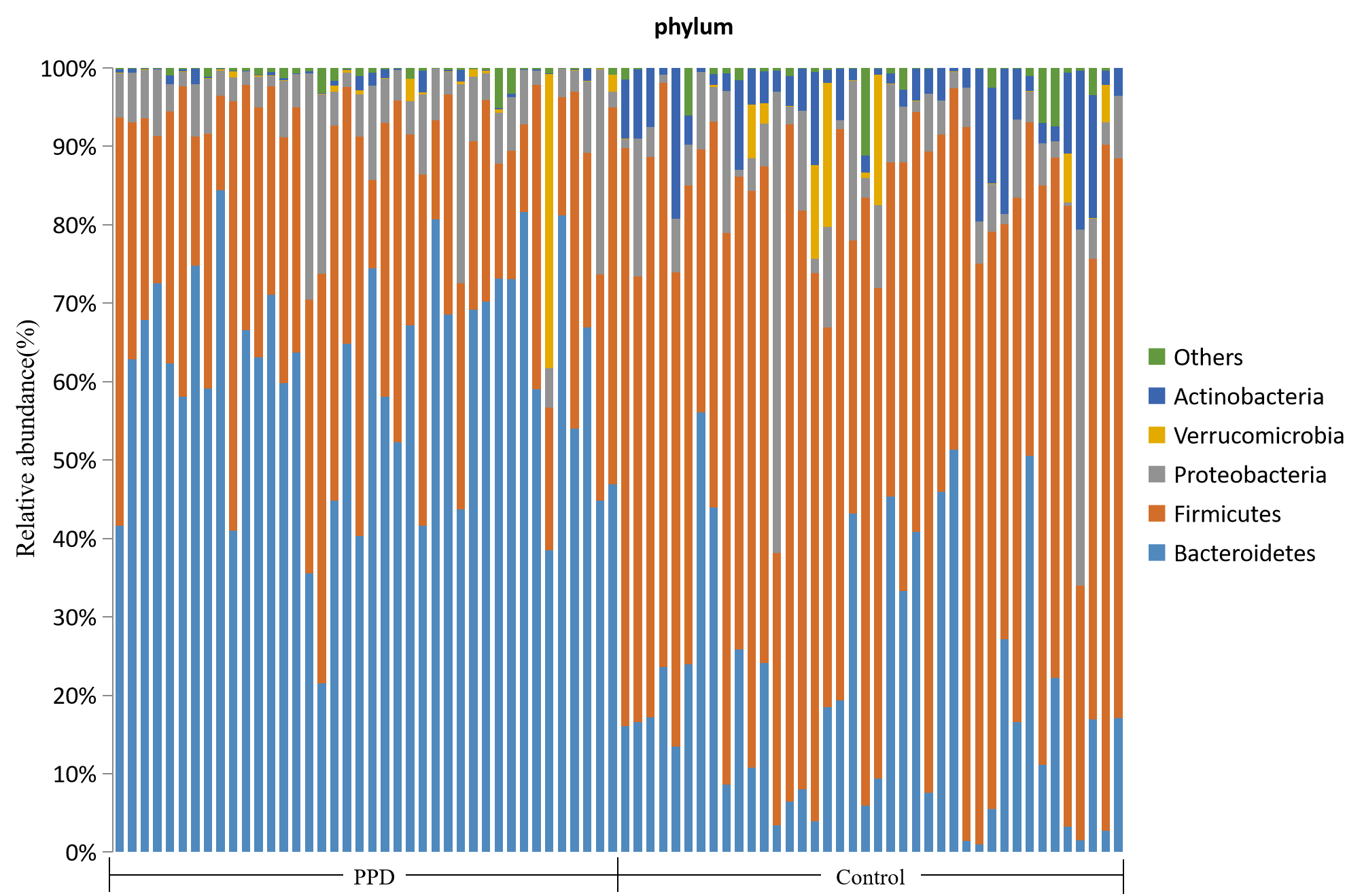

Supplement: Supplementary Figure 2 — Stacked bar plots showing the distribution of taxa in PPD patients and healthy controls. Relative abundance at the phylum level. [file Image_2.PNG]

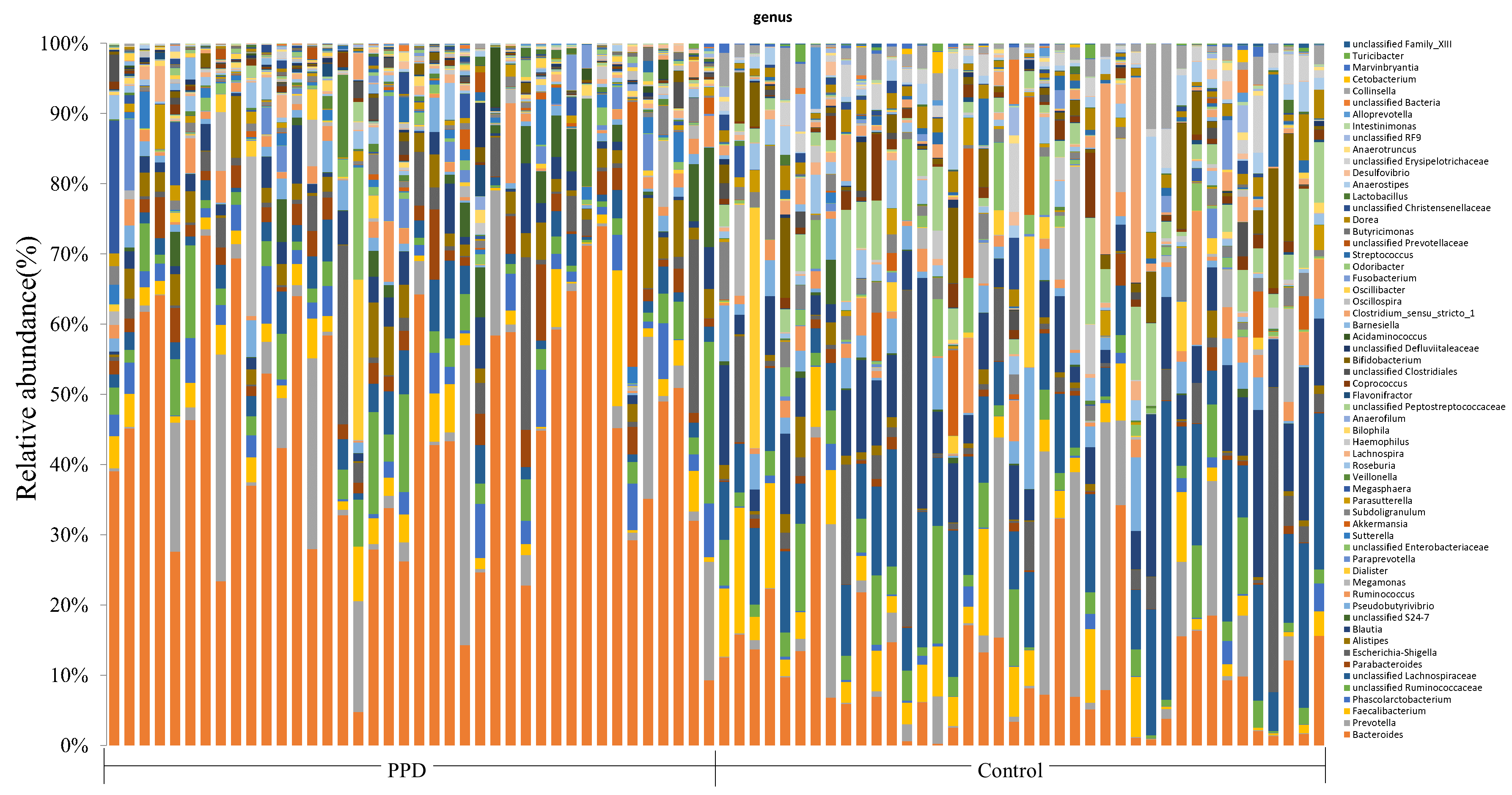

Supplement: Supplementary Figure 3 — Stacked bar plots showing the distribution of taxa in PPD patients and healthy controls. Relative abundance at the genus level. [file Image_3.PNG]

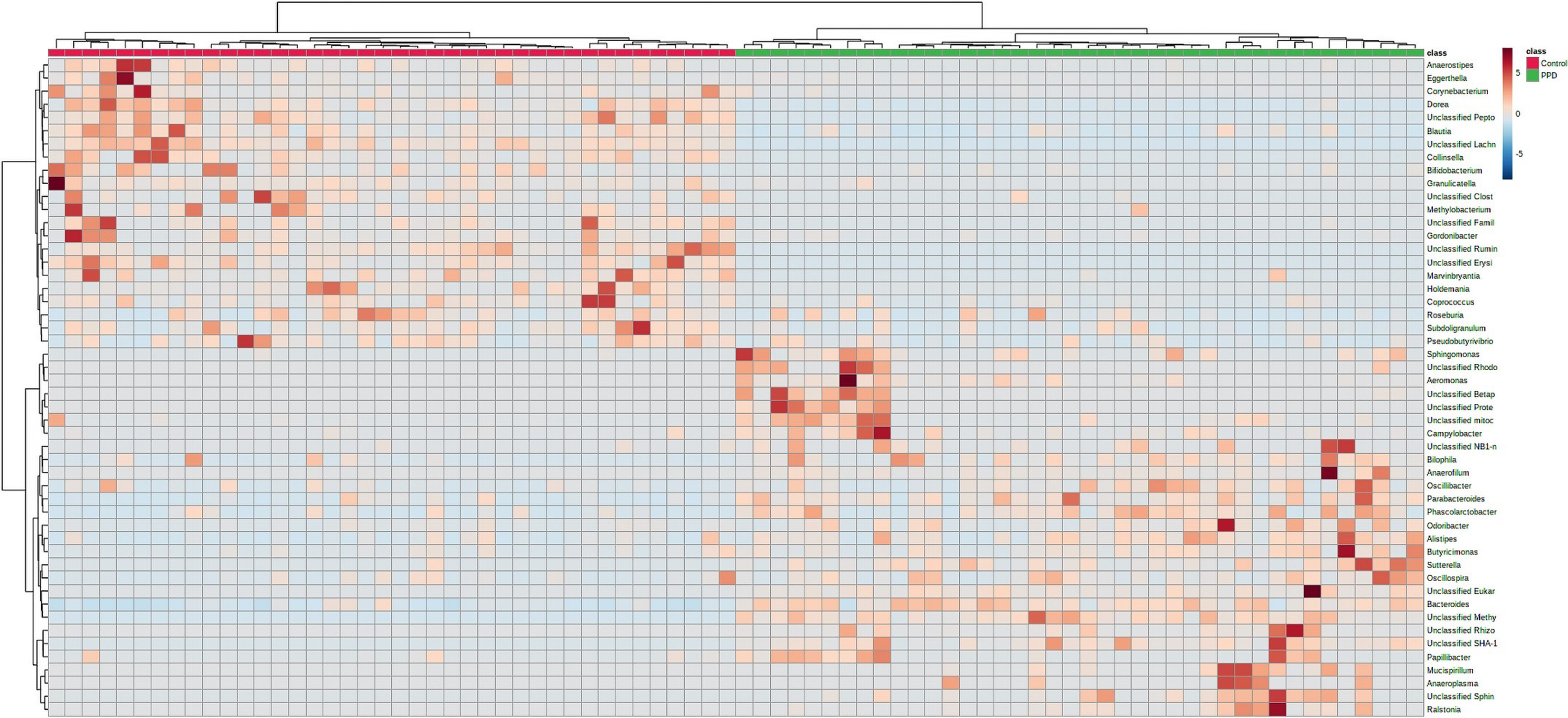

Supplement: Supplementary Figure 4 — Cluster analysis and heatmap of top 50 species abundance at the genus level in PPD patients and healthy controls. The clustering result is shown in the form of a dendrogram and heatmap. Metaboanalyst (version 5.0) was used for clustering. Each column represents the analyzed samples, and each row represents one of the top 50 microbiota genera. On top of the heatmap: red squares, healthy controls; green squares, PPD patients. The dendrogram for samples is shown on top of the heatmap, and the microbiome dendrogram is on the left side. Dark blue to dark red color gradient denotes lower to higher expression. [file Image_4.JPEG]
