# Supplementary material for: Altered gut microbiota profile in patients with perimenopausal panic disorder
Source: Front Psychiatry. 2023 May 25;14:1139992. doi: 10.3389/fpsyt.2023.1139992 (PMC10249373; doi:10.3389/fpsyt.2023.1139992)
Supplement: Supplementary Table 1 — Top five phyla microbiota between PPD patients and healthy controls. Median (IQR: 25, IQR: 75). [file Table_1.docx]

Supplement Table 1

The top 5 phyla microbiota between PPD and Healthy Controls. Median (IQR25, IQR75)

|  | Control (n=40) | PPD (n=40) | *P* value |
| --- | --- | --- | --- |
| Bacteroidetes * | 16.76 (6.72, 26.83) | 62.96 (45.35, 70.86) | <0.05 |
| Firmicutes * | 64.78 (50.16, 73.80) | 30.77 (19.47, 39.41) | <0.05 |
| Proteobacteria | 5.174 (2.191, 9.946) | 5.468 (3.241, 7.178) | 0.765 |
| Verrucomicrobia * | 0.02 (0.0029, 0.077) | 0.066 (0.023, 0.37) | 0.012 |
| Actinobacteria * | 3.792 (1.899, 8.502) | 0.156 (0.071, 0.451) | <0.05 |

Note: * mean *p* value＜0.05
