# Supplementary material for: Altered gut microbiota profile in patients with perimenopausal panic disorder
Source: Front Psychiatry. 2023 May 25;14:1139992. doi: 10.3389/fpsyt.2023.1139992 (PMC10249373; doi:10.3389/fpsyt.2023.1139992)
Supplement: Supplementary Table 2 — Top 60 genera microbiota between PPD patients and healthy controls. [file Table_2.docx]

Supplement Table 2

The top 60 genera microbiota between PPD and Healthy Controls.

|  | Control (N=40) | | PPD (N=40) | | |  |
| --- | --- | --- | --- | --- | --- | --- |
|  | Median | (Q1, Q3) | Median | | (Q1, Q3) | *P* value |
| Bacteroides | 9.380 | (4.078, 15.47) | | 43.926 | (27.745, 58.767) | ＜0.05 |
| Prevotella | 0.846 | (0.149, 6.066) | | 1.280 | (0.526, 4.656) | 0.117 |
| Faecalibacterium | 3.460 | (2.092, 7.487) | | 2.297 | (1.209, 4.106) | 0.033 |
| Phascolarctobacterium | 0.235 | (0.084, 1.018) | | 2.429 | (0.996, 4.845) | ＜0.05 |
| unclassified Ruminococcaceae | 2.943 | (1.66, 4.782) | | 2.974 | (1.443, 5.277) | 0.893 |
| unclassified Lachnospiraceae | 10.350 | (6.933, 16.38) | | 2.449 | (1.483, 4.1997) | ＜0.05 |
| Parabacteroides | 0.316 | (0.107, 1.009) | | 2.109 | (1.404, 3.354) | ＜0.05 |
| Escherichia-Shigella | 0.861 | (0.399, 3.927) | | 0.749 | (0.355, 3.022) | 0.736 |
| Alistipes | 0.553 | (0.147, 1.163) | | 2.618 | (1.604, 4.377) | ＜0.05 |
| Blautia | 7.043 | (4.099, 9.97) | | 1.552 | (0.761, 3.735) | ＜0.05 |
| unclassified S24-7 | 0.033 | (0.006, 0.244) | | 0.112 | (0.033, 4.248) | 0.002 |
| Pseudobutyrivibrio | 2.892 | (0.940, 4.456) | | 0.865 | (0.289, 1.737) | 0.001 |
| Ruminococcus | 1.438 | (0.512, 3.381) | | 1.181 | (0.340, 2.123) | 0.175 |
| Megamonas | 0.067 | (0.0188, 0.669) | | 0.064 | (0.023, 1.0098) | 0.806 |
| Dialister | 0.065 | (0.012, 0.585) | | 0.080 | (0.026, 0.544) | 0.453 |
| Paraprevotella | 0.018 | (0.003, 0.276) | | 0.080 | (0.0187, 1.505) | 0.011 |
| unclassified Enterobacteriaceae | 0.454 | (0.102, 2.042) | | 0.216 | (0.0995, 1.1267) | 0.166 |
| Sutterella | 0.003 | (0.00028, 0.0201) | | 0.557 | (0.168, 1.645) | ＜0.05 |
| Akkermansia | 0.020 | (0.00296, 0.0771) | | 0.066 | (0.0211, 0.370) | 0.015 |
| Subdoligranulum | 1.840 | (0.564, 2.933) | | 0.409 | (0.0865, 0.928) | ＜0.05 |
| Parasutterella | 0.110 | (0.0202, 0.463) | | 0.210 | (0.0802, 1.001) | 0.078 |
| Megasphaera | 0.006 | (0.0021, 0.0165) | | 0.058 | (0.0188, 0.1735) | ＜0.05 |
| Veillonella | 0.052 | (0.0217, 0.172) | | 0.170 | (0.061, 0.5001) | 0.002 |
| Roseburia | 1.062 | (0.5032, 2.767) | | 0.435 | (0.203, 0.915) | 0.005 |
| Lachnospira | 0.247 | (0.093, 0.636) | | 0.255 | (0.1085, 0.677) | 0.736 |
| Haemophilus | 0.124 | (0.032, 0.4825) | | 0.077 | (0.0282, 0.272) | 0.331 |
| Bilophila | 0.019 | (0.00593, 0.0673) | | 0.285 | (0.120, 0.673) | ＜0.05 |
| Anaerofilum | 0.000 | (0, 0.00254) | | 0.050 | (0.0151, 0.113) | ＜0.05 |
| unclassified Peptostreptococcaceae | 2.876 | (1.712, 5.584) | | 0.070 | (0.0239, 0.167) | ＜0.05 |
| Flavonifractor | 0.038 | (0.0171, 0.103) | | 0.095 | (0.0568, 0.2088) | 0.003 |
| Coprococcus | 0.860 | (0.3803, 1.5) | | 0.138 | (0.0587, 0.4898) | ＜0.05 |
| unclassified Clostridiales | 0.050 | (0.0152, 0.71) | | 0.070 | (0.0261, 0.392) | 0.447 |
| Bifidobacterium | 0.921 | (0.2149, 5.921) | | 0.095 | (0.0272, 0.402) | ＜0.05 |
| unclassified Defluviitaleaceae | 0.074 | (0.04062 0.277) | | 0.146 | (0.0949, 0.291) | 0.160 |
| Acidaminococcus | 0.000 | (0, 0) | | 0.012 | (0.0035, 0.032) | ＜0.05 |
| Barnesiella | 0.024 | (0.0096, 0.1659) | | 0.022 | (0.008, 0.125) | 0.810 |
| Clostridium_sensu_stricto_1 | 0.681 | (0.2036, 1.577) | | 0.039 | (0.0094, 0.1112) | ＜0.05 |
| Oscillospira | 0.025 | (0.00856, 0.0891) | | 0.117 | (0.0329, 0.3507) | ＜0.05 |
| Oscillibacter | 0.055 | (0.0179, 0.1175) | | 0.176 | (0.0694, 0.483) | 0.001 |
| Fusobacterium | 0.008 | (0.00176, 0.0483) | | 0.023 | (0.0072, 0.0945) | 0.069 |
| Odoribacter | 0.035 | (0.00738, 0.103) | | 0.133 | (0.0126, 0.445) | 0.003 |
| Streptococcus | 0.402 | (0.1764, 0.8003) | | 0.064 | (0.0293, 0.155) | ＜0.05 |
| unclassified Prevotellaceae | 0.000 | (0, 0.00123) | | 0.002 | (0, 0.0085) | 0.005 |
| Butyricimonas | 0.017 | (0.0024, 0.0589) | | 0.136 | (0.0286, 0.297) | ＜0.05 |
| Dorea | 1.357 | (0.7876, 2.303) | | 0.072 | (0.0372, 0.2019) | ＜0.05 |
| unclassified Christensenellaceae | 0.084 | (0.0140, 0.4984) | | 0.036 | (0.0073, 0.249) | 0.115 |
| Lactobacillus | 0.038 | (0.0075, 0.118) | | 0.027 | (0.0075, 0.132) | 0.954 |
| Anaerostipes | 0.825 | (0.4066, 2.353) | | 0.035 | (0.0182, 0.1101) | ＜0.05 |
| Desulfovibrio | 0.007 | (0.00174, 0.1099) | | 0.026 | (0.0099, 0.134) | 0.039 |
| unclassified Erysipelotrichaceae | 1.532 | (0.4491, 2.556) | | 0.045 | (0.0224, 0.106) | ＜0.05 |
| Anaerotruncus | 0.055 | (0.0133, 0.272) | | 0.019 | (0.00634, 0.049) | 0.011 |
| unclassified RF9 | 0.013 | (0.00434, 0.254) | | 0.011 | (0.00177, 0.0351) | 0.117 |
| Intestinimonas | 0.076 | (0.0168, 0.2112) | | 0.032 | (0.0191, 0.1101) | 0.144 |
| Alloprevotella | 0.004 | (0, 0.0252) | | 0.006 | (0, 0.1141) | 0.965 |
| unclassified Bacteria | 0.018 | (0.0068, 0.0382) | | 0.019 | (0.00415, 0.0475) | 0.693 |
| Collinsella | 1.441 | (0.4447, 3.24) | | 0.015 | (0.0031, 0.0435) | ＜0.05 |
| Cetobacterium | 0.002 | (0, 0.0101) | | 0.000 | (0, 0) | ＜0.05 |
| Marvinbryantia | 0.134 | (0.0627, 0.3848) | | 0.000 | (0, 0.00314) | ＜0.05 |
| Turicibacter | 0.060 | (0.0198, 0.1728) | | 0.002 | (0, 0.01064) | ＜0.05 |
| unclassified Family_XIII | 0.043 | (0.0144, 0.1529) | | 0.010 | (0.0035, 0.0249) | ＜0.05 |
