# Supplementary material for: Altered gut microbiota profile in patients with perimenopausal panic disorder
Source: Front Psychiatry. 2023 May 25;14:1139992. doi: 10.3389/fpsyt.2023.1139992 (PMC10249373; doi:10.3389/fpsyt.2023.1139992)
Supplement: Supplementary Table 3 — Correlation analysis between BMI, PASS, PDSS, HAMA scales, and fecal microbiota. [file Table_3.docx]

Supplement Table 3

Correlation analysis between BMI, PASS, PDSS, HAMA Scales and fecal microbiota.

|  | BMI | | PASS | | PDSS | | HAMA | |
| --- | --- | --- | --- | --- | --- | --- | --- | --- |
|  | r | *p* value | r | *p* value | r | *p* value | r | *p* value |
| Bacteroides | -0.002 | 0.992 | -0.150 | 0.354 | 0.377 | 0.017 | 0.308 | 0.053 |
| Faecalibacterium | 0.224 | 0.165 | -0.045 | 0.782 | -0.282 | 0.078 | -0.322 | 0.042 |
| Phascolarctobacterium | 0.363 | 0.021 | 0.358 | 0.023 | 0.181 | 0.263 | 0.265 | 0.098 |
| Parabacteroides | 0.204 | 0.208 | 0.394 | 0.012 | 0.107 | 0.509 | 0.095 | 0.560 |
| Alistipes | 0.109 | 0.504 | 0.366 | 0.020 | 0.322 | 0.043 | 0.466 | 0.002 |
| Blautia | 0.019 | 0.905 | 0.260 | 0.106 | 0.120 | 0.462 | -0.140 | 0.389 |
| Pseudobutyrivibrio | -0.037 | 0.821 | -0.009 | 0.958 | 0.159 | 0.328 | 0.064 | 0.696 |
| Paraprevotella | 0.012 | 0.939 | 0.058 | 0.722 | -0.043 | 0.793 | 0.044 | 0.787 |
| Sutterella | 0.037 | 0.822 | -0.103 | 0.528 | -0.136 | 0.402 | 0.089 | 0.586 |
| Akkermansia | 0.161 | 0.322 | 0.313 | 0.049 | 0.062 | 0.702 | 0.037 | 0.820 |
| Subdoligranulum | 0.023 | 0.888 | 0.188 | 0.246 | -0.111 | 0.495 | 0.118 | 0.469 |
| Megasphaera | 0.286 | 0.074 | 0.088 | 0.591 | -0.128 | 0.432 | -0.073 | 0.654 |
| Veillonella | -0.164 | 0.311 | -0.045 | 0.782 | 0.225 | 0.162 | 0.250 | 0.120 |
| Roseburia | 0.303 | 0.057 | 0.151 | 0.351 | 0.020 | 0.904 | -0.101 | 0.534 |
| Bilophila | 0.148 | 0.363 | 0.042 | 0.797 | 0.151 | 0.351 | 0.308 | 0.053 |
| Flavonifractor | 0.146 | 0.369 | -0.069 | 0.672 | 0.042 | 0.796 | 0.165 | 0.310 |
| Coprococcus | 0.082 | 0.616 | 0.028 | 0.862 | -0.402 | 0.010 | -0.191 | 0.237 |
| Bifidobacterium | -0.052 | 0.752 | 0.257 | 0.109 | 0.185 | 0.254 | 0.215 | 0.184 |
| Clostridium_sensu_stricto_1 | -0.100 | 0.539 | -0.084 | 0.605 | -0.035 | 0.830 | -0.148 | 0.362 |
| Oscillospira | -0.253 | 0.115 | 0.050 | 0.760 | 0.097 | 0.550 | 0.052 | 0.750 |
| Oscillibacter | 0.146 | 0.367 | 0.200 | 0.216 | -0.125 | 0.443 | 0.266 | 0.098 |
| Odoribacter | -0.199 | 0.218 | 0.111 | 0.494 | -0.025 | 0.877 | 0.306 | 0.055 |
| Streptococcus | -0.170 | 0.293 | 0.036 | 0.824 | 0.103 | 0.526 | 0.327 | 0.040 |
| Butyricimonas | -0.092 | 0.571 | 0.220 | 0.172 | 0.015 | 0.929 | 0.266 | 0.097 |
| Dorea | 0.269 | 0.093 | 0.019 | 0.906 | -0.021 | 0.899 | -0.193 | 0.233 |
| Anaerostipes | 0.016 | 0.923 | 0.045 | 0.783 | -0.061 | 0.710 | -0.070 | 0.666 |
| Desulfovibrio | -0.223 | 0.168 | 0.079 | 0.628 | -0.008 | 0.962 | 0.095 | 0.561 |
| Anaerotruncus | 0.180 | 0.268 | 0.228 | 0.158 | -0.197 | 0.223 | 0.019 | 0.908 |
| Collinsella | 0.077 | 0.636 | 0.187 | 0.248 | -0.118 | 0.468 | 0.080 | 0.625 |
| Turicibacter | -0.069 | 0.672 | -0.003 | 0.988 | -0.086 | 0.598 | -0.078 | 0.634 |
